# A new method for the estimation of minimum adult frog density from a large-scale audial survey

**DOI:** 10.1038/s41598-020-65560-6

**Published:** 2020-05-25

**Authors:** Andris Čeirāns, Aija Pupina, Mihails Pupins

**Affiliations:** Daugavpils University, Institute of Life Sciences and Technologies, address: Vienības iela 13, Daugavpils, LV-5401 Latvia

**Keywords:** Ecology, Zoology

## Abstract

Audial surveys of anuran amphibians (frogs, toads and similar) are cost-effective and allow for the coverage of large areas, but they are usually regarded as unsuitable for population size estimations due to imperfect detection. Our study demonstrated a method for obtaining minimum adult population size estimates from vocalising anuran counts by using sex ratios, life history and vocalising behaviour parameters from other studies. We collected data from 2016 to 2018 for seven taxa on 65 plots (each 25 km^2^) representing the entirety of Latvia. Among taxa, average breeding waterbody audible detection probabilities ranged from 0.56 to 0.88 per plot, minimum adult frog density (*MAFD*) estimates were from 12.0 to 51.7 individuals per km^2^, but the estimated fraction of population covered by *MAFD* varied from 57 to 86%. The least accurate density estimates were in taxa with brief calling activity and quiet mating calls (*Rana temporaria*), and in taxa with a calling activity dependent on the numbers of males in a pond (*Bufo bufo*). Our study suggests that lek-breeders would be more suitable than explosive-breeding taxa for minimum population size estimates from audial data. The use of *MAFD* allowed for coarse minimum population size estimates for the entire country from the audial monitoring data, these ranged from 3.7 ± 0.5 thousand (*Bombina bombina*) to 1.64 ± 0.47 million (*B.bufo*) adults.

## Introduction

Knowledge of population size is crucial for evaluating the future prospects of a population, making conservation management decisions, and selecting management priorities for protected areas. Anuran amphibian (frogs, toads, and similar) monitoring programs from different parts of the world have relied on surveys of male vocalisations to detect and monitor the status of anuran populations over time^1–3^. Their metrics are based on the male calling intensity rather than the calling male numbers, because the later is often difficult to estimate from a distance. Examples of the metrics used include vocalising intensity categories of the amphibian calling index^3^, call latency (time between the survey start and the first detected call)^4^, call counts from automated recorders^5^, and simply the presence or non-detection of a species^6,7^.

Audial surveys for anurans are cost-effective because they allow for the coverage of large areas, including sites that are not accessible or are problematic for other types of surveys like mark-recapture or visual encounter. However, audial surveys are usually regarded as less accurate than other methods for population estimations. Several studies that paired acoustic abundance estimates with mark-recapture surveys supported the assertion that audial surveys are well-correlated with abundance^8–10^, whereas other studies indicated that they are not informative at low densities^11^ or that the calling index is weakly related to anuran abundances^12^. The reliability of vocalising male counts varies among species and depends on the time of the visit^7,13,14^. One study demonstrated the possibility of using calling male counts for the estimation of the true breeding population size when most of the males in the local population were calling simultaneously^8^. However, vocalisation data has never been used as a main source of true adult population size estimates due to imperfect detection of all individuals in the population and uncertainty of the relationship between the calling male metrics and the total population size^7,14–16^.

There are no logical preconditions against the estimation of adult anuran population size from a calling male count, if we know the following parameters: (1) sex ratio, (2) proportion of males vocalising at the survey time, and (3) the proportion of the total adult male population, present in a waterbody at the time of observation (for the mainly terrestrial species, which do not use waterbodies as permanent habitats). The last two parameters are very sensitive to survey timing. For instance, few calling males may indicate (i) a low vocalising activity, (ii) that most males are in terrestrial habitats and therefore absent from a breeding waterbody, or (iii) a small population size. The clarification of the three parameters above is time- and labour-consuming for each population. However, for population size estimates, it is possible to count calling males at peak breeding activity and use average peak detection probabilities and sex ratios from other studies. Such an approach may lead to incorrect results for each particular waterbody due to natural variation of parameters; for instance, sex ratios may vary between sites and years^5,17,18^. However, for a large-scale study with many sites, concordance between averages from other studies and those in a given survey is a logical assumption. During a monitoring survey with many sites and relatively few visits to each site, it is unlikely that all the sites will be visited at the perfect time since the maximum activity period can be brief and the frog calling activity can be unpredictable. This means that the real number of males present most likely will be higher than those estimated from the calling male counts and, hence, we will estimate only the minimum adult population size. This is still important information for nature conservation purposes, where population size overestimations carry risks for not taking the necessary conservation measures. If the sampling is representative, it is possible to extrapolate the data and to acquire minimum population density or population size for a larger territory.

We conducted a large scale survey of vocalising amphibians from 2016 to 2018, as a part of an amphibian and reptile monitoring program that was managed by the Nature Conservation Agency of Latvia. The survey covered the whole territory of the country; we visited more than 1 200 waterbodies near the peak calling activities of multiple species and documented more than 13 000 calls. The main metric recorded in the field was the number of calling males, but the vocalising intensity categories of calling index from^3^ were used as supplementary information for the evaluation of calling activity. The aim of the present study was to evaluate a new method for estimating the minimum adult frog density (*MAFD*) from calling anuran counts, to identify possible sources of estimation errors and to demonstrate its use in nature conservation.

## Results

### Survey results

We recorded a total of seven taxa [hybridogenic water frog species complex containing the Pool Frog (*Pelophylax lessonae*), the Edible Frog (*P. esculentus*), and the Marsh Frog (*P.ridibundus*) (the later was absent on our plots), were treated together as Water Frogs *Pelophylax* spp.] during the study (Table 1). *Pelophylax* spp. was the most commonly registered taxon in all measures – number of plots, calling individuals per waterbody, percentage of occupied waterbodies. This taxon had highest breeding waterbody detection probability (WDP) in audial surveys (0.88 on average), and high inter-year breeding waterbody fidelity (IYF) estimate (0.82 on average). The lowest WDP in audial surveys (0.56 on average) was found in the Common Frog (*Rana temporaria*). The Fire-Bellied Toad (*Bombina bombina*) and the Common Spade-Foot Toad (*Pelobates fuscus*) had the lowest IYF, which on average were 0.68 and 0.73 per plot, respectively (Table 1). The contribution from a most productive survey (CMPS) was higher in taxa breeding late in the season – *B.bombina*, the European Tree-Frog (*Hyla arborea* species group), and *Pelophylax* spp. (Table 1). In spite of the small sample size, a positive relationship between the number of calling males and the proportion of occupied waterbodies was best supported in *H.arborea*, compared to other taxa (Table 2). The later is interesting for implications for the conservation state of *H.arborea* in Latvia (see Discussion).

**Table 1:** Synopsis of the audial surveys (average ± standard deviation (sample size)) in 2016–2018 at 65 plots throughout Latvia.

Taxon name	Fraction of plots with audial records	Calling males per waterbody^a^	Fraction of waterbodies with audial records	WDP^b^	IYF^c^	CMPS^d^
*Bombina bombina*	0.09	3.3 ± 1.0 (6)	0.26 ± 0.21 (6)	no data	0.68 ± 0.17 (4)	0.90 ± 0.11 (4)
*Pelobates fuscus*	0.29	3.3 ± 2.7 (19)	0.14 ± 0.09 (19)	no data	0.73 ± 0.24 (6)	0.77 ± 0.23 (5)
*Bufo bufo*	0.97	2.8 ± 1.6 (63)	0.31 ± 0.19 (63)	0.69 ± 0.24 (25)	0.83 ± 0.18 (21)	0.81 ± 0.12 (22)
*Hyla arborea*	0.09	5.3 ± 2.7 (6)	0.31 ± 0.22 (6)	no data	no data	0.90 ± 0.07 (3)
*Rana arvalis*	0.80	5.7 ± 4.9 (52)	0.20 ± 0.14 (51)	0.78 ± 0.15 (9)	0.78 ± 0.19 (9)	0.79 ± 0.17 (11)
*Rana temporaria*	0.81^e^	4.3 ± 3.0 (53)	0.24 ± 0.18 (53)	0.56 ± 0.32 (25)	0.74 ± 0.20 (7)	0.87 ± 0.12 (5)
*Pelophylax* spp.	0.98	5.7 ± 2.4 (64)	0.52 ± 0.15 (64)	0.88 ± 0.09 (23)	0.82 ± 0.13 (22)	0.88 ± 0.09 (31)

^a^only for waterbodies with records; ^b^or waterbody detection probability for audial surveys; ^c^or inter-year fidelity in breeding site use; ^d^or a contribution from the most productive survey to the total number of calling males given year; ^e^0.84 when include plots with only visual observations.

**Table 2:** Statistics for the Poisson Regression between the average number of calling males per waterbody (dependent variable) and the proportion of occupied waterbodies on plot (quantitative factor) in the present study (statistically significant at P < 0.001).

Taxon	df	Coefficient at quantitative factor in the equation	Chi-Square	Pseudo R^2^ adj (%)
*Bombina bombina*	5	−0.012	78	50
*Pelobates fuscus*	18	−0.0049	10	<1
*Bufo bufo*	62	0.0096	663	13
*Hyla arborea*	5	0.025	696	89
*Rana arvalis*	51	0.022	4256	24
*Rana temporaria*	52	0.0041	138	1
*Pelophylax* spp.	63	0.011	1321	17

### Calling activity

With the exception of *H.arborea* (small sample size), the average earliest vocalisation dates varied by less than 10 days between taxa (Table 3). The longest seasonal ranges of calling activity were in *Pelophylax* spp., *B.bombina*, and the Common Toad (*Bufo bufo*); the first two were observed to vocalise occasionally until early September (unpublished data) – well after our survey end dates in mid-June. Other taxa showed a length of the vocalisation season on average of less than one calendar month (Table 3). Although most taxa had statistically significant trends in the numbers of calling males during the breeding season, they strongly varied among years and were well supported only in some cases (Table 4). Maximum effect of observation date was in *R.temporaria*, where Pseudo R^2^ adj explained 50% of variation in 2018 (compared to 36% in the Moor Frog (*Rana arvalis)* and 3% in *B.bufo* that year, see Supplement).

**Table 3:** Calling activity start date and duration (average ± standard deviation) in seven taxa observed in our study in 2016–2018.

Taxon	Earliest record date (n = 3)	Calling activity range, days (n = 3)
*Bombina bombina*	14 April (±10 days)	40 ± 21
*Pelobates fuscus*	14 April (±8 days)	22 ± 5
*Bufo bufo*	7 April (±6 days)	50 ± 13
*Hyla arborea*	15 May (±3 days)	23 ± 12
*Rana arvalis*	7 April (±5 days)	27 ± 5
*Rana temporaria*	7 April (±5 days)	28 ± 5
*Pelophylax* spp.	16 April (±7 days)	63 ± 16

**Table 4:** Statistics for the Poisson Regression between the average number of calling males per waterbody (dependent variable) and the relative date after onset of the breeding season (quantitative factor) in seven taxa observed in our study in 2016–2018 (2016–2017 for *H.arborea*) (average ± standard deviation).

Taxon	Fraction of years with significant relationship^a^	df	Trend	Chi-Square	Pseudo R^ 2^ adj (%)
*Bombina bombina*	0.7	6 ± 0	Positive	83 ± 81	13 ± 2
*Pelobates fuscus*	1.0	9 ± 3	Varies^b^	102 ± 81	11 ± 2
*Bufo bufo*	1.0	40 ± 12	Negative	144 ± 81	4 ± 3
*Hyla arborea*	0.5	5	Positive	10	1
*Rana arvalis*	1.0	25 ± 11	Negative	1102 ± 461	18 ± 17
*Rana temporaria*	1.0	22 ± 6	Negative	1123 ± 611	32 ± 18
*Pelophylax* spp.	1.0	42 ± 1	Positive	810 ± 893	11 ± 11

^a^at *P* = 0.0016 in *H.arborea* and *P* < 0.001 in other taxa, ^b^negative in two and positive in one year, for details see Supplement.

**Figure 1:**
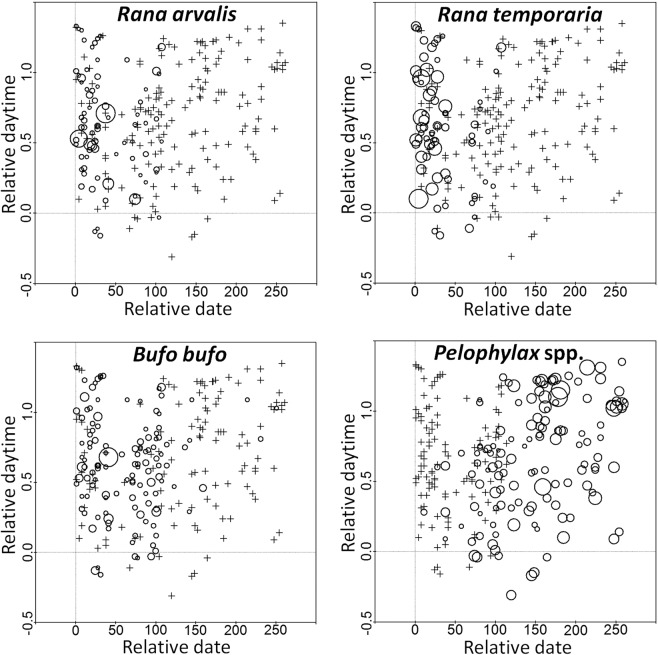
Plots illustrating the daily and seasonal calling activity patterns in the four most common taxa observed during 298 audial surveys within 65 plots throughout Latvia in 2016–2018. The size of a circle corresponds to the average number of calling males per waterbody (only for those with records of the given taxon in given survey; minimum value of 1.0 in all taxa, maximum value of 50.0 in *R.arvalis*, 12.6 in *R.temporaria*, 20.0 in *B.bufo*, 14.6 in *Pelophylax* spp.); the crosses represents surveys with no records for the given taxon; x axis – decimally-transformed survey dates where 0 is the date of onset of any anuran taxa calling activity in the given year; y axis – decimally-transformed time of a day where 0 is astronomic noon, but 1 – sunset in the given date.

Poisson Regression Analyses indicated very poor relationships between the number of calling males and the time of the day, with Pseudo R^2^ adj typically less than 10%, and their trends (negative vs positive) varied between years for the same taxa (see Supplement). Four the most common taxa had data covering the whole range of the calling period, from early morning until late night (rare taxa that were present in few plots had insufficient data). None had an apparent daily activity pattern (Fig. 1). In the early season, *Pelophylax* spp. were recorded only in daylight surveys, but showed no marked male count patterns in the mid or late season. In some taxa (*B.bufo, R.arvalis*), the highest number of calling males were registered during few daytime surveys early in the season, which could still be a coincidence (see Discussion).

### Population density estimations

**Figure 2:**
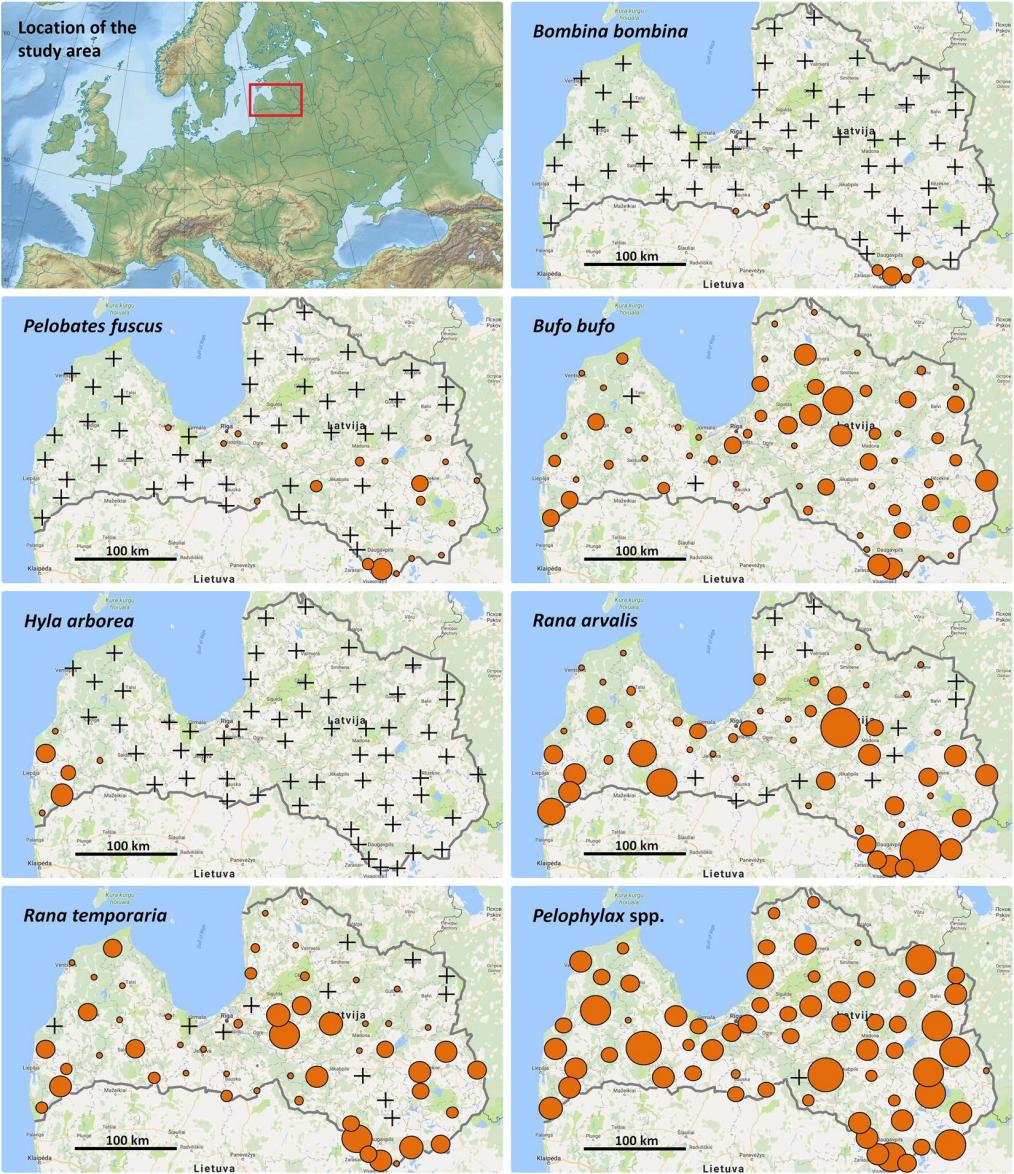
Study area location map and maps of estimated densities of calling males (*Dc*) for seven anuran taxa observed in anuran audial surveys at 65 plots (circles) throughout Latvia in 2016–2018. The size of a brown circle is proportional to the density of the given taxon (circle size in all taxa corresponds to same density range, from <2 to >40 calling males/km^2^); cross marks indicate absence of a taxon in the surveys. Images are based on maps from https://www.mapas-del-mundo.net/ (the study area location) and *Google Maps* (taxa densities).

Plots with high or low densities were geographically clustered for all taxa, and their patterns varied (Fig. 2). Averages calling male densities (*Dc* metric) were highest in *Pelophylax* spp., but highest minimum adult frog densities (*MAFD* metric) were in *R.arvalis*; we estimate that our *MAFD* calculations from audial-only data in various taxa covered from 57% (in *R.temporaria*) to 86% (in *R.arvalis*) of the population (Table 5). The values of *Dc* and *MAFD* were higher for plots with higher WDP; that relationship was true for all taxa with relevant data and was very similar for both metrics (as for *Dc* metric: *B.bufo* df = 24, Chi-Square = 1700, Pseudo R^2^ adj = 17%, *R.arvalis* df = 8, Chi-Square = 4638, Pseudo R^2^ adj = 67%, *R.temporaria* df = 22, Chi-Square = 4313, Pseudo R^2^ adj = 32%, *Pelophylax* df = 22, Chi-Square = 4210, Pseudo R^2^ adj = 38%; *P* < 0.001 in all the taxa).

**Table 5:** The estimated densities for calling males (*Dc)* and the minimum adult population (*MAFD*), and the estimated part of the total population that was detected audibly and covered by *MAFD* estimates (average ± standard deviation; median (sample size)), for seven taxa observed in anuran audial surveys at 65 plots throughout Latvia in 2016–2018.

Taxon	*Dc*, individuals/km^2^	*MAFD*, individuals/km^2^	Fraction of population covered by *MAFD* estimates
*Bombina bombina*	4.1 ± 4.5; 2.8 (6)	12.0 ± 13.5; 7.8 (6)	no data
*Pelobates fuscus*	2.1 ± 2.9; 0.9 (19)	17.0 ± 20.0; 9.0 (19)	no data
*Bufo bufo*	3.8 ± 4.2; 2.6 (63)	40.7 ± 45.1; 27.8 (63)	0.65 ± 0.21; 0.67 (25)
*Hyla arborea*	6.8 ± 7.1; 5.1 (6)	29.5 ± 31.0; 22.1 (6)	no data
*Rana arvalis*	7.2 ± 9.9; 3.5 (52)	51.7 ± 71.9; 25.2 (52)	0.86 ± 0.15; 0.90 (9)
*Rana temporaria*	5.2 ± 5.6; 2.9 (53)	36.4 ± 39.0; 20.8 (53)	0.57 ± 0.25; 0.63 (21)
*Pelophylax* spp.	10.3 ± 8.4; 7.1 (64)	35.2 ± 29.3; 25.6 (64)	0.78 ± 0.13; 0.78 (14)

Small waterbodies accounted for the largest part of the population in all taxa; for *B. bombina* and *P.fuscus*, they were used by more than 90% of the total population (Table 6). The largest contribution from a medium sized waterbodies was in *R.arvalis*, where 30% of its population was found. Ditches were more important in *Rana* frogs, especially in *R.temporaria*, relative to the other taxa. Large waterbodies accounted for less than 5% of the population for all taxa on average. In the semi-aquatic taxa (*B.bombina, Pelophylax*) and in *P.fuscus* the estimated population densities were positively affected by overall waterbody availability, while for early-spring breeders (*Bufo, Rana* spp.) and *H.arborea* it was a less important or negligible factor (Table 7).

**Table 6:** Relative contributions of the four waterbody types to minimum adult frog density (*MAFD*) estimates for the given taxon (average ± standard deviation) at 65 plots throughout Latvia in 2016–2018.

Taxon (plots)	Small lentic waterbodies (<0.5 ha)	Medium lentic waterbodies (0.5–10.0 ha)	Large lentic waterbodies (>10.0ha)	Ditches
*Bombina bombina* (6)	0.93 ± 0.11	0.06 ± 0.09	0	0.01 ± 0.02
*Pelobates fuscus* (19)	0.93 ± 0.23	0.07 ± 0.23	0	0
*Bufo bufo* (63)	0.65 ± 0.33	0.23 ± 0.30	0.02 ± 0.09	0.09 ± 0.21
*Hyla arborea* (6)	0.75 ± 0.23	0.22 ± 0.23	0.01 ± 0.02	0.02 ± 0.05
*Rana arvalis* (52)	0.54 ± 0.37	0.30 ± 0.36	0.03 ± 0.12	0.13 ± 0.25
*Rana temporaria* (53)	0.65 ± 0.38	0.18 ± 0.29	0.01 ± 0.02	0.17 ± 0.33
*Pelophylax* spp. (64)	0.72 ± 0.25	0.20 ± 0.23	0.03 ± 0.09	0.06 ± 0.12

**Table 7:** Statistics for the Poisson Regression between the estimated densities of calling males (or *Dc* metric) (dependent variable) and the total number of available waterbodies on plot (quantitative factor) in the present study (statistically significant at *P* < 0.001 in all taxa).

Taxon	df	Coefficient at quantitative factor in the equation	Chi-Square	Pseudo R^2^ adj (%)
*Bombina bombina*	5	0.0059	1775	92
*Pelobates fuscus*	18	0.0044	2023	39
*Bufo bufo*	61	0.0013	925	4
*Hyla arborea*	5	0.00061	19	<1
*Rana arvalis*	50	0.0017	2604	5
*Rana temporaria*	52	0.0025	4188	15
*Pelophylax* spp.	62	0.0028	12250	34

## Discussion

Plots with higher or lower densities formed distinct geographical clusters. Their pattern varied among taxa (Fig. 2) indicating that the observed variation was related to true regional differences in population sizes and not to survey effort. Plots with higher breeding waterbody audial detection probabilities (WDP) had higher estimated densities (*Dc* and *MAFD*) in all the taxa; this may indicate that results we obtained still include some population underestimations caused by insufficient sampling in some of the plots.

In *Pelophylax* spp., *H.arborea* and *B.bombina*, the most productive audial survey accounted for about 90% from the total score (CMPS of 0.88-0.90 in Table 1), but for the most of the spring-breeding terrestrial species (i.e. *B.bufo, P.fuscus, R.arvalis*) its contribution was lower (CMPS of 0.77-0.81). In the later group of taxa, male migration to or from breeding sites between surveys is very likely, because they may have populations breeding at different times. For instance, small populations may breed later than large ones^19^. *R.temporaria* had higher CMPS (0.87) than other early-spring breeders (i.e. *R.arvalis*, *B.bufo*), and its audial records demonstrated most a notable decline in male numbers during the breeding period compared to other taxa; this may indicate that few newcomers arrive at breeding sites after the first wave of breeding adults.

In Latvia, early spring breeders (*R.temporaria, R.arvalis, B.bufo*) start to breed at the same time of year, in contrast to other parts of their range with milder climates where there are some time lags between these taxa^20,21^. The most productive time for the first survey in the present study was the first week after the onset of any calling activity, and the optimal time for the last survey was the warm period of late spring or the beginning of summer. One or two surveys conducted over 5–10 day intervals after the first survey were necessary for the later-breeding *Rana* and *Bufo* populations and for *P.fuscus*.

For the population size estimates, data from different years were not pooled. This was justified by estimations of inter-year fidelity in breeding waterbody use (IYF); our surveys in a most productive year detected calling on average at only 68–83% of sites with confirmed calling in any year, depending on taxon (Table 1). The lowest IYF was in *B.bombina, P.fuscus, R.temporaria* which can be explained by their association with a small lentic waterbodies or ditches. The conditions of these habitats are more prone to inter-year climate variations and often artificial modifications and their use by amphibians may depend on their quality in a given year. 

We did not test the accuracy of the published peak activity parameters and sex ratios that could be a source of significant population size estimation errors. Errors may also be introduced during estimation of the size of the sampled area, because distances over which calls can be heard may vary depending on the landscape, weather, time of day, and observer^22^. Magnitude of their effect remains unknown, but they would be less likely to occur in small waterbodies, which were fully within observers’ hearing range. Hence, in taxa, where the small waterbodies contributed to the main part of population estimates (e.g., *B. bombina* and *P. fuscus* in the present study), the results should be more accurate than in taxa with a smaller contribution from these habitats.

Our data did not suggest that the daily time for surveys was very important for calling male detection. This finding was unexpected given that male calling activity often depends on the time of day^20^. The use of individual male counts rather than activity indices would lessen or compensate for daily calling activity differences, because individuals making few calls with low activity may lead to the same score as chorusing males. Correct selection of the time of day should still be important in some taxa that typically have calling activity confined to the night-time, such as *H.arborea*^23,24^. In our study, surveys within that taxon’s limited range in Latvia were conducted only after sunset, and hence our data may not show the importance of survey-time selection for *H.arborea*.

In the audial surveys, two taxa had notably lower detectability than the others. *R.temporaria* had the lowest WDP (0.56) and the smallest fraction of population covered by *MAFD* estimation (0.57). This species is a truly explosive breeder (sensu^25^), arriving with the onset of anuran breeding activities and starting to spawn immediately^26,27^. Its calling activity declines quickly during a short breeding period (e.g., 28 d in present study) and, therefore, the timing of the field survey is crucial for the adequate detection of this species. The audial detection of *R.temporaria* is made more difficult by its quiet call, which is easily obscured by obstacles and natural background noises, especially during the daytime. In warm weather, this frog vocalises both day and night (day choruses are weaker), but under cool weather conditions, *R.temporaria* vocalises mostly after dark^20^, when surveys are not always possible. In comparison, the other “brown frog” (*Rana*) species, *R.arvalis*, had a much higher detectability (WDP of 0.78 and coverage by *MAFD* estimates of 0.86) due to its less variable calling activity according to time of day^20^ and smaller effect from decline in calling activity during the breeding season (data from the present study). Apart from detectability issues, a possible source for the underestimation of *R.temporaria* population could be the omission of waterbodies that were not visible on orthophoto maps, such as periodically water-filled relief depressions in forests, where *R.temporaria* is the most common breeding amphibian species^28^.

The other taxon with less accurate results in the audial surveys was *B.bufo*, in which only 65% of the true population was detected audibly. In this species, the proportion of vocalising males is negatively correlated to the size of the breeding group, because in large groups, more males switch from calling to actively searching for females^29^. The sex ratio in this species is always strongly biased towards males^30,31^ and so far, behaviour switching has not been described in other taxa with less biased sex ratios. This variation in vocalising male proportions can produce inaccurate audial survey data for population size estimates. However, such estimates can still be in line with the minimum population size concept if they are samples from large or different-sized populations. In large groups, population size will be underestimated, and in small groups it will be overestimated, but the error for large groups will have a stronger effect on the results. The presence of an observer usually has little effect on *B.bufo* behaviour. The skewed sex ratio and mating behaviour of the species mean that free-moving females are almost absent and the visual male counts can provide a more precise estimation of the adult population than audial surveys.

Territorial behaviour, associated with ‘lek-mating’ [sensu^25,32^], is found in *Bombina*^33^, *H.arborea* species complex^34^ and *Pelophylax*^35^. All three taxa had high CMPS estimates (having close to maximum scores during the most productive survey) and good waterbody detection probabilities. This suggests that lek-breeders may be more suitable than explosive breeders for population size estimates from audial surveys.

Our calculation method allows for rough estimations of the total anuran population sizes for the whole state. Such estimations are important for nature conservationists and national authorities, since European Union member states are obliged to give the European Commission a report every six years about the progress made in the implementation of the Habitats Directive, and these reports must also have population size estimates for a whole member state, including all the taxa from our study except *B.bufo*. Medians are the most appropriate measure of central tendency in this study since their use removes the impacts of inadequately low values, originating from surveys with low calling activity, and very high values, originating from overestimated chorus sizes. The potential species range in Latvia for most taxa is roughly 60,000 km^2^ (the total area of the state is 64,589 km^2^). Using medians for *MAFD* metrics (Table 5), taking into account the percentage of plots where taxa were present (Table 1) and the standard deviations of parameters (Table 8), we estimate that the whole state yields at least 157 ± 23 thousand of adult *P.fuscus*, 1.62 ± 0.47 million *B.bufo*, 1.51 ± 0.43 million *Pelophylax* spp., 1.21 ± 0.19 million *R.arvalis* and 1.05 ± 0.22 million *R.temporaria* (the later and *B.bufo* still considerably underestimated; see above).

**Table 8:** Parameters used in *MAFD* calculation formula (Eq. 2) (average ± SD, if more than one estimate available (sample size)); Ma - the proportion of males in a waterbody vocalising during the peak activity, Mp - the proportion of the adult males from total population present in waterbodies in the peak of the breeding season, Ms - the proportion of males in total population.

Taxon name	Ma	Mp	Ms	Sources
*Bombina bombina*	0.61	1.00 ± 0.00 (2)	0.56 ± 0.07 (3)	^36,44–46^
*Pelobates fuscus*	0.29	0.65 ± 0.04 (2)	0.55 ± 0.04 (5)	^43,52–56^
*Bufo bufo*	0.18	0.78 ± 0.08 (2)	0.66 ± 0.09 (3^a^)	^29,31,57^
*Hyla arborea*^*b*^	0.58	0.72	0.55 ± 0.04 (2)	^17,58^
*Rana*^*c*^	0.30	0.95 ± 0.05 (2)	0.48 ± 0.06 (7)	^26,42,56,59–61^, and unpublished data
*Pelophylax*^d^	0.63	1.00 ± 0.00 (2)	L 0.47 ± 0.14 (2) E 0.36 ± 0.08 (2)	^39,62–64^

^a^All in the same source^31^; ^b^for northern populations of *Hyla arborea* species complex^41^; ^c^Combined for *Rana arvalis* and *R.temporaria*; ^d^in *Ms* column L - *P.lessonae*, E - *P.esculentus*, in our calculations, we used a simplification inferred from published data on L and E presence depending on waterbody size^63,65^: L sex ratio for small waterbodies and ditches, E – for large waterbodies, and (L + E)/2 – for medium-sized waterbodies.

*B.bombina* is only found in south and south-eastern Latvia and has a species range of ~470 km^2^ ^36^. It was recorded in all surveyed plots within its range, and minimum *B.bombina* population size in Latvia could be estimated as only 3.7 ± 0.5 thousand adults. This is a rare and protected species, whose density in the present study was positively correlated to waterbody availability. Hence, this could be an important factor affecting local population size, and the creation of new breeding waterbodies within the species’ range could be essential for the increase of this species population in Latvia.

*H.arborea* is a newly established taxon in Latvia, originating from the release of 4,110 juveniles from 1988 to 1992 in a site in the south-west of the country^37^. The present range of this population covers ~4,000 km^2^ in Latvia (Andris Čeirāns, unpublished data) and ~800 km^2^ in north-western Lithuania[^38^] and two records in flickriver.com (sites Pašile and Drupiai; G.Gražulevičius photos)]. *H.arborea* was recorded in all plots within the species range, although male density in the central part of the range was 6 to 84-fold greater than that on the periphery of the range. The present population of *H.arborea* in Latvia could be estimated as at least 88 ± 7 thousand adults. In this species, large populations with many males per breeding site occupied more waterbodies, waterbody availability did not affect population size, and there was a large difference in population density between central and peripheral parts of the range. All the above may indicate an expanding *H.arborea* population in Latvia and north-west Lithuania that has not yet colonised all the suitable habitats and territories.

Our study indicates that the *MAFD* estimation method used can be a useful tool for nature conservationists and scientists. All the main metrics currently used in vocalising anuran monitoring [e.g. in^3–5^] are variations of the registration of calling intensity, not male counts, and they can be weakly related to true population sizes, especially at low densities^11,12^. Our *Dc* and *MAFD* metrics are based on male numbers, and may produce similar results under the various calling activity levels of individual males. Unlike calling intensity based metrics, these metrics would be more accurate at low and medium densities, when individual calls can easily be counted, and less accurate at high densities with chorusing frogs when individual calls can be distinguished only by very close approach to the breeding site.

## Materials and Methods

### Study site selection

Our study was carried out on 65 plots (Fig. 2). Each plot was a 5 × 5 km square in the Transverse Mercator Projection (TMP). For the selection of 60 plots, we divided the whole territory of Latvia into 10 similar-sized TMP sectors, and randomly selected 5–7 plots in each, proportionally to the size of terrestrial part in a given sector. We also added five more randomly selected plots within the range of *B.bombina*, an EU Habitat Directive Annex II species, along the southern and south-eastern border of the country, because all the other random plots were outside the range of this species. Hence, the survey was semi-representative for the whole country.

In each plot we randomly selected waterbodies from orthophoto maps proportionally to (i) their presence in open versus closed landcover type, (ii) spatial distribution in four equal parts of a plot (determined as four quadrants over plot along N–S and E–W axes), and (iii) their numbers in three size categories – small (less than 0.5 ha in surface area), medium-size (0.5–10.0 ha), and large (more than 10.0 ha). We surveyed 15–35 (mean 18.6 ± SD 4.5) waterbodies and 5–10 points (6.0 ± 1.6) near larger ditches (i.e. channels for soil drainage and water flow; excludes channelled river or stream stretches with flowing water) per plot. Most of the waterbodies (91%) present on plots were small; we sampled 27% of them, and also 49% of all the medium-size, and 57% of all the large waterbodies. For waterbody dimension measurements we used orthophoto maps in the Google Earth Pro program (Google LLC, Mountain View, California, U.S.).

### Field surveys

To cover peak calling activities of all anuran taxa, we visited each plot at least once during the each of the following periods: (i) April 1–15, (ii) April 16–30, and (iii) May 20–June 10. We selected days with warmest weather forecasts within given periods, when we expected high calling activity^20,39,40^. If we observed low activity (a calling index^3^ value of 1) or only few sites with call records, we repeated the survey at the given plot some days later or in the same calendar period in the following year. Surveys were not confined to certain times of day or night, except for the surveys in six plots in the range of *H.arborea* (a species complex ^41^) in south-western Latvia, which were visited only after the sunset, because this taxon typically does not vocalise in daylight^23,24^. We conducted a total of 298 calling surveys in 2016-2018, on average 4.6 ± SD 1.1 (range 3–7) surveys per plot.

Typically, only one observer per survey was present due to the large amount of fieldwork and the limited numbers of trained observers. During surveys the observer recorded the number of calling males at locations near waterbodies or ditches over a period of at least 5 minutes (no maximum time limit). After this, whenever it was feasible due to the condition of waterbody banks and the time of the day, the observer approached and walked around or along the waterbody or ditch for a better counting of calling males, and for counting visible breeding adults and egg clumps or strings. In some cases, with chorusing frogs, when counts were hindered by overlapping calls or banks were inaccessible and did not allow approaches close enough to distinguish individual calls, the number of calling males was recorded as an average between the minimum and maximum estimates by the observer. Observers also recorded the time of day, air temperature, wind speed (Beaufort scale), and sky conditions (clear, overcast, partial clouds) during the onset, middle and end of the survey, to confirm the overall suitability of the survey conditions.

### Data processing and statistics

We used the largest number of simultaneously counted males per visit in a given site (waterbody or ditch) for population density estimations. Data from multiple years were not pooled; if we had data from several years for the same plot, we used counts only from the year with the best record, because we observed whole choruses to move between neighbouring waterbodies in consecutive years. For plots with data from several years, we estimated inter-year fidelity (IYF) in the use of breeding waterbodies. This was calculated as a ratio of waterbodies with a call record in the most productive year to waterbodies with a call record in pooled data for all years. IYF included both, true breeding site shifts and false shifts from undetected waterbodies, which we did not discriminate in our estimation.

There were many cases when the most productive survey in terms of total counted males was the most productive only for the part of the individual sites of a given plot. We estimated the contribution from a most productive survey (CMPS) as a ratio of the most productive survey score to the total calling male score in a given year. Lower CMPS values would indicate the desirability for more surveys per breeding period, and high CMPS values that fewer surveys are sufficient for data collection.

We also calculated the probability of detecting a breeding waterbody from the audial surveys (WDP) as a ratio of waterbodies with calling male records to waterbodies with any evidence of the presence of adults during the breeding period (including both, audial records and direct (adults seen) or indirect (presence of spawn, tadpoles, metamorphs) visual evidence) for a given taxon in a given year.

In activity analyses, we transformed calendar dates into decimal numbers. In order to remove inter-year variation in breeding season start dates, we further transformed acquired decimals into relative dates after the earliest audial record of any taxon in a given year. We also transformed time of day on the middle of the survey into decimal numbers and then into relative daytime, where 0 was the astronomic noon and 1 was the sunset time on a given date, taken from World Weather Online web-site (worldweatheronline.com, accessed 05.01.2019.). This was done to compensate for the increase in day length during the sampling period.

Our data did not fit normal distribution, so we used Poisson Regression Analysis to test statistical significance of relationships between dependent variables, such as calling males per waterbody, *Dc* or *MAFD* and various quantitative factors from our data sets. Data was transformed into counts by multiplying them by factor of 100 and rounding to integers. Our date and daytime effect analyses were done separately for each year, and here we used only data from surveys with records of a given taxon. Other relationships we studied in pooled data sets from all years. We performed all statistical analyses using Statgraphics Plus 5.0 (Statgraphics Technologies, Inc., The Plains, Virginia, U.S.) and SPSS PASW Statistics 18 (IBM Corporation, Armonk, New York, U.S.). XY(Z) charts or ‘bubble plots’ were prepared in CANOCO for Windows 4.5 software (Biometris –Plant Research International, Wageningen, The Netherlands).

### Calculation of metrics

We introduced two new metrics (*Dc* and *MAFD*) to quantify frog populations. The density of calling males (*Dc*) is basically a waterbody category-specific extrapolation of recorded calling frogs to all the plot waterbodies, and the summation of results from all the categories given per area unit. In each waterbody category we used the total calling male score and the ratio of total to surveyed habitat in a plot. Small waterbodies (<0.5 ha) showed no correlation between their size and the number of calling males of any taxa (Generalised Linear Models, *P* > 0.1 in all cases), were fully within the hearing range of an observer and therefore could be treated as units in our ratio estimations. In larger waterbodies in the study area frogs typically breed only in the shallow shoreline zone (unpublished data), so there may be a correlation between the length of a shoreline and population size. Often only part of a medium-size or large waterbody is within the hearing range of an observer so in our ratio estimations we treated them as linear shoreline habitat.

The *Dc* metric was calculated as follows (Eq. 1):

1 $$Dc=\frac{{\sum }_{i}\frac{C\ast Pt}{Ps}}{S}$$

where *Dc*- is the density of calling males; *Ʃ* – represents the summation of the calculations for four waterbody categories (*i*): small (<0.5 ha), medium (0.5–10.0 ha), large (>10.0 ha) waterbodies, and ditches; *C*- is the number of calling males scored in a waterbody category *i*; *Pt* – total habitats in a waterbody category *i*, which is the number of all small waterbodies on the plot, or total length of waterbody perimeters for medium or large waterbodies, or length of all ditches; *Ps* – surveyed habitats in a waterbody category *i*, which is the number of sampled small waterbodies, the total length of the perimeters sampled for medium or large waterbodies, or total sampled ditch length; *S* – is the area of the plot.

The sampled length of waterbody perimeter or ditch was the length within the hearing range of an observer. The distance from which it is possible to hear a call depends on taxon, weather, habitat, obstacles and background noise^22^. Unless count results indicated larger distances, in calculations we used approximate minimum hearing distances of 500 m for *Pelophylax* spp. and *H.arborea*, 150 m for *B.bufo*, 100 m for *R.arvalis* and *B.bombina*, and 50 m for *R.temporaria* and *P.fuscus*. Such a distances were approximations of average values estimated from first-year surveys using GPS coordinates of calling sites, most distant points, where calls were audible and distance measurements by Google Earth Pro.

The second metric (*MAFD*) transfers scores of calling males into a proxy for the true adult frog population. It relies on the probability of detecting males with reference to their breeding behaviour patterns (presence in breeding waterbody and probability of being heard) to estimate their true population and sex ratios to transfer male population estimates into total adult population. Since this metric is based on maximum detection probabilities with perfect survey timing and environmental conditions (which are unlikely to be met in a random survey) the estimate would typically be smaller than the true population so we obtain the minimum adult frog population density estimate (or *MAFD* metric).

The *MAFD* metric was calculated as follows (Eq. 2):

2 $$MAFD=\frac{Dc}{Ma\ast Mp\ast Ms}$$

where *MAFD* – the minimum density of adult frogs on plot; *Dc* – the density of the calling males; *Ma* – the proportion of males that are vocalising during the peak activity (only from males present in waterbodies); *Mp* – the proportion of male total population present in waterbodies in the peak of the breeding season; *Ms* – the proportion of males in population (i.e., sex ratio). Taxon-specific parameters *Ma, Mp, Ms* were taken mainly from published studies (Table 8). Information on these parameters was generally scarce; thus, we did not find published *Ma* data on *B.bombina* and *Rana*. For these taxa we used *Ma* values from our unpublished visual observations of several ponds with excellent view of the water surface and a highly transparent water column. Regarding *Ms*, not all the adult females reproduce every year, and in terrestrial taxa (i.e. taxa not using waterbodies outside breeding season) the operational sex ratio at breeding waterbodies differs from the sex ratio in the whole adult population^31,42^. There are very limited data available for how much the operational sex ratio differs from the real sex ratio in surveyed taxa, so we ignored possible inter-species differences. We did reduce the published operational male proportions in terrestrial taxa - *P.fuscus*, both *Rana*, and *H.arborea*, by 15% (which was an average difference in^31,43^ for *B.bufo* and *P.fuscus*) to transform them into real proportions (i.e. *Ms*). For *B.bufo* we used published data on real sex ratio^31^. We did not find published data on *B.bombina* sex ratios and therefore we used information on other European *Bombina* species^44–46^.

We compared our *MAFD* estimates to visual observation records. In 58% of the plots at least one taxon had sufficient data from visual counts when they covered at least 75% of the surveyed waterbodies in the breeding season of a given taxon. We applied to observed frogs the following detection probabilities, which were inferred from other studies^47–50^ and personal observations: 0.75, when there were no obstacles to visual surface or underwater observations and active breeding behaviour (vocalising, moving, and mating) was observed; 0.50, when there were no obstacles to surface observations, but underwater visibility was poor or active behaviour absent; 0.20, when there were strong obstacles to visual observation; 0.10, for separate casual observations. The detection probability for egg masses was set as 0.90^51^. Egg counts were transferred into the respective numbers of males using the sex ratios from Table 8, and we also used sex ratios for visually counted adult *Pelophylax* spp. frogs with unknown sex (in contrast, typically only males are free moving in breeding aggregations of *Bufo* and *Rana*^29^, pers.obs.). We used *Ma* to transfer visual data into virtual calling males for each waterbody and then added undetected virtual males to waterbody calling male counts and used Eqs. 1 and 2 to obtain the new density estimate (*NDE*). The fraction of population covered by an *MAFD* estimate was calculated as the ratio of *MAFD* to *NDE*.

## Supplementary information

Supplementary Information.
